# Crosslinking Rapidly Cured Epoxy Resin Thermosets: Experimental and Computational Modeling and Simulation Study

**DOI:** 10.3390/polym15051325

**Published:** 2023-03-06

**Authors:** Ahmed Al-Qatatsheh, Jaworski C. Capricho, Paolo Raiteri, Saulius Juodkazis, Nisa Salim, Nishar Hameed

**Affiliations:** 1 School of Engineering, Swinburne University of Technology, Melbourne, VIC 3122, Australia; 2 School of Molecular and Life Sciences, Faculty of Science and Engineering, Curtin University, Perth, WA 6845, Australia; 3 Optical Sciences Centre and ARC Training Centre in Surface Engineering for Advanced Materials (SEAM), Swinburne University of Technology, Melbourne, VIC 3122, Australia

**Keywords:** solvate ionic liquid, rapid curing, the intrinsic reaction coordinates (IRCs) method, molecular dynamics

## Abstract

The power of computational modeling and simulation for establishing clear links between materials’ intrinsic properties and their atomic structure has more and more increased the demand for reliable and reproducible protocols. Despite this increased demand, no one approach can provide reliable and reproducible outcomes to predict the properties of novel materials, particularly rapidly cured epoxy-resins with additives. This study introduces the first computational modeling and simulation protocol for crosslinking rapidly cured epoxy resin thermosets based on solvate ionic liquid (SIL). The protocol combines several modeling approaches, including quantum mechanics (QMs) and molecular dynamics (MDs). Furthermore, it insightfully provides a wide range of thermo-mechanical, chemical, and mechano-chemical properties, which agree with experimental data.

## 1. Introduction

Rapidly cured epoxy-resin thermoset polymers provide futuristic alternatives for the additive manufacturing of a wide range of applications in space, defense, and medtech, to name a few [1,2]. In addition to their relatively low cost, they can provide high processability with “on-demand” properties when controlling their cure rate or lowering their cure temperature. Still, this process is iterative-based, requiring complex experiments under an extremely controlled environment to achieve the expected outcomes. A viable and more feasible approach is adopting the computational modeling and simulation approach, which allows researchers to conduct a computer-based simulated set of experiments, saving materials, time, and effort and reducing waste.

Computational chemistry software packages based on quantum mechanics are powerful and can provide many properties at the atomic level. However, current applications are limited to a certain number of atoms that cannot, in most cases, provide a reliable representation of the physical system under study. On the other hand, molecular dynamics simulation is growing widely among researchers who usually utilize this approach to investigate material properties on the atomic scale. Several studies concerning the molecular modeling of epoxy-thermosets have been published using crosslinking modeling to investigate superior mechanical properties [3,4,5,6,7,8,9]. Still, inconsistency in molding procedures and outcomes has been observed due to more than one aspect. At first, modeling success usually depends on the spatial cutoff criterion between the pre-defined activated ends of the molecules capable of forming crosslinks. Therefore, this success will depend on identifying the right activated end, which may become challenging when running molecular dynamic simulations to predict innovative material properties [10]. Equally, the crosslinking approach should aim to check cutoff distances while applying a well-defined set of relaxation cycles coupled with the molecular computations until crosslinking is achieved and then carefully cooling the crosslinked samples at room temperature. In the literature, these steps have rarely been fulfilled completely. Nonetheless, deciding upon the algorithms for generating the targeted structure may affect predicting the required properties. For instance, both single-step and multi-step approaches may lead to comparable thermal and mechanical properties, but both can significantly differ in molecular weight distribution [4]. Likewise, the lack of full understanding of the reaction kinetics of the crosslinked system can severely impact the calculation of the targeted properties, especially when crosslinking goes through several phases similar to ours or others, such as epoxy-anhydride hardener [11]. Another common source of inconsistency is the partial automatic charges required to run molecular simulations; hence, a reliable and detailed approach is needed to ensure accuracy, which, in return, is not always comprehensively published, leading to increased uncertainty [12]. Furthermore, the equilibration of the crosslinked models with the confirmation of liquid equilibration before crosslinking is highly required to ensure that the reaction mixture’s potential energy and density reach stability [4]. Additionally, choosing the right size for the computationally modeled system is crucial for reproducibility and reliability since building models with small sizes may cause misrepresentation of the real-life system, leading to misleading results. Likewise, using a statistically representative number of simulation samples is critical in order to capture the average behaviors of the epoxy-thermosets [13].

**Figure 1:**
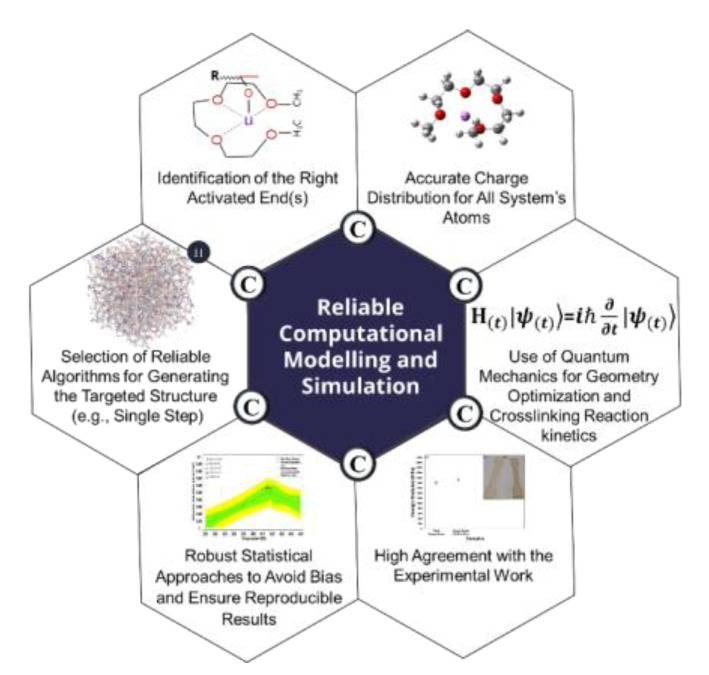
Summary of all factors we considered in our protocol to overcome most of the abovementioned challenges leading to inconsistency.

In this study, we followed quantum mechanics and molecular dynamics approaches to investigate the properties associated with the rapid cure, providing a roadmap to overcome most of the abovementioned challenges leading to inconsistency (Figure 1). We also present the first reliable and systematic approach for the molecular modeling of a crosslinked polymer using a solvate ionic liquid (SIL). We implemented this approach to the generation of 3D crosslinked samples of the epoxy precursor Diglycidyl Ether of Bisphenol A (DGEBA), the curing agent 4,4′-methylenedianiline (MDA), and the SIL based on lithium bis(trifluoromethanesulfonyl) imide ([Li] [TFSI]) in triglyme (G3) [1]. Additionally, we utilized molecular modeling outcomes to predict the thermo-mechanical properties of the polymer, including glass transition temperature (Tg) and Young’s modulus.

## 2. Materials and Methods

### 2.1. Materials

#### Sample Preparation

The synthesis of the epoxy precursor diglycidylether of bisphenol A (DGEBA), curing agent 4,4′-methylenedianiline (MDA), lithium bis(trifluoromethanesulfonyl)imide, and ligand was reported elsewhere [1]. All materials were obtained from Sigma (Melbourne, Australia) and were used as received.

### 2.2. Methods

#### 2.2.1. Experimental Work

**Figure 2:**
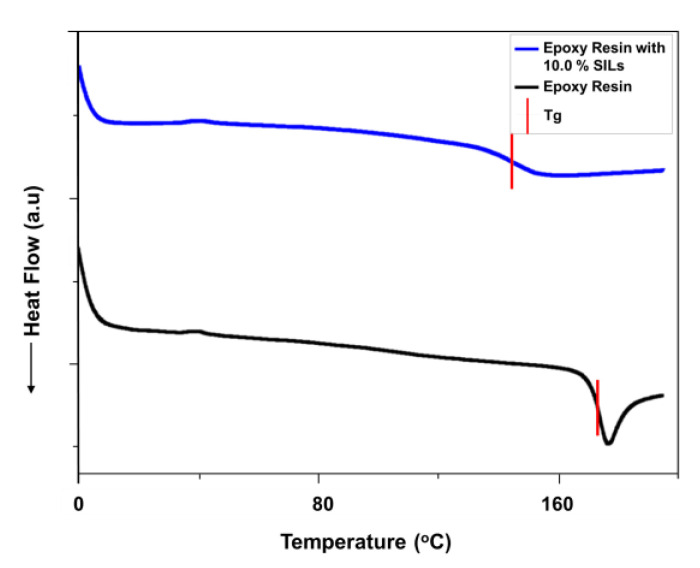
DSC curves of samples with/without 10.0 wt.% SIL.

- Phase behavior and thermo-mechanical properties:

The epoxy-resin samples with 10.0 wt.% additive were prepared, while the epoxy-resin control samples were prepared without additives. The phase behavior investigation of both sets was performed using differential scanning calorimetry (DSC) using 5–10 mg of each sample under an atmosphere of nitrogen gas with a TA–DSC model Q200 instrument. All samples were heated from 25 °C to 250 at 20 °C/min. The glass transition temperatures of the epoxy resin control and 10.0 wt.% SIL samples were 172 °C and 144 °C, respectively, as illustrated in Figure 2.

**Figure 3:**
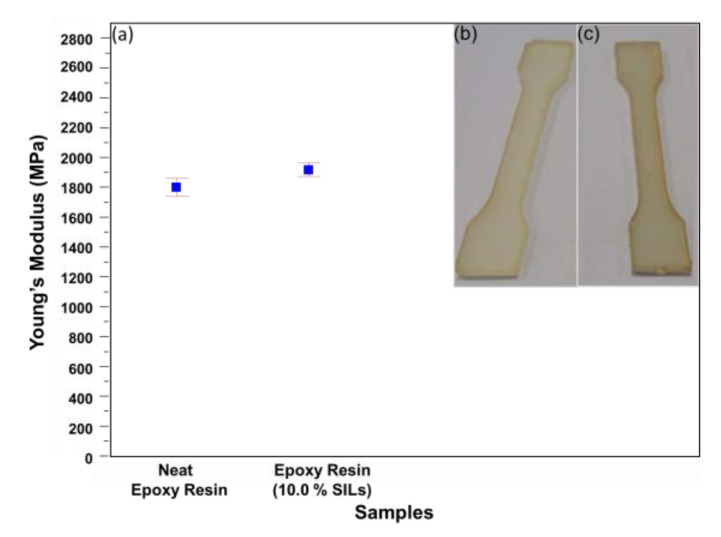
Young’s modulus: (**a**) statistical significance analysis (*p* < 0.05), where values are in blue and error bars in red, (**b**,**c**) neat epoxy resin and epoxy resin with 10.0 wt.% SIL samples.

The composites’ tensile properties, particularly Young’s modulus, were measured using the Instron 30 kN SD tensile testing machine. All tensile tests were performed with dog-bone-shaped specimens in line with the ASTM D638 standard, and no less than five specimens were tested to obtain the average values. Figure 3 shows a statistically significant difference between the average values of Young’s modulus of the neat epoxy resin samples and values of samples with the 10.0 wt.% SIL. Young’s modulus of samples of the neat epoxy resin and the epoxy resin with 10.0 wt.% SIL was 1.75 ± 0.06 GPa and 1.86 ± 0.04 GPa, respectively.

#### 2.2.2. Protocol

**Figure 4:**
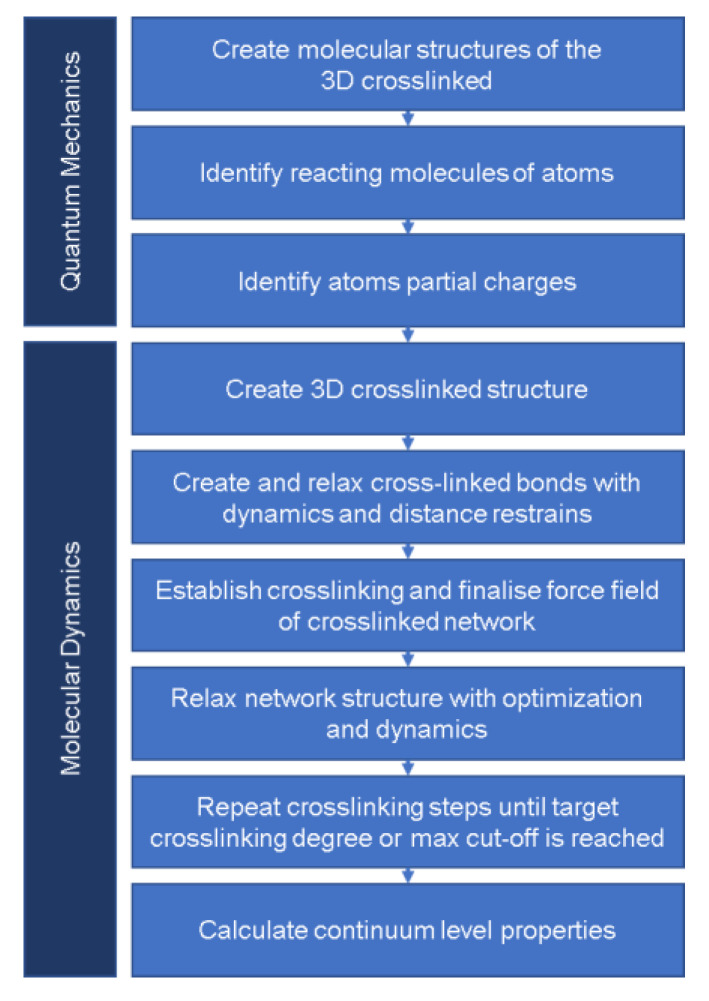
Summary of computational modeling protocol followed to calculate continuum-level properties of the 3D crosslinked polymer.

Figure 4 illustrates the protocol we developed, from the initial step of creating the molecular structures until calculating continuum-level properties.

- Atomic Structure

**Figure 5:**
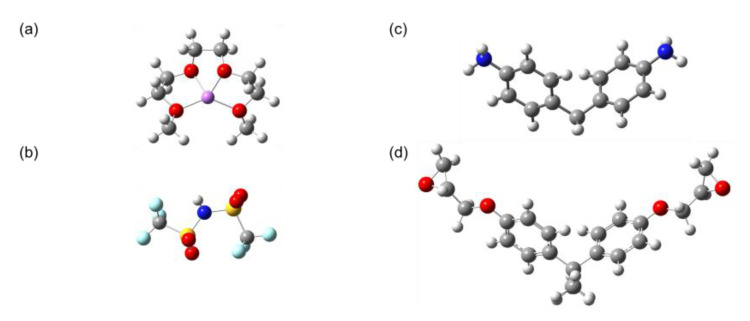
Initial atomic structure of the components of the polymer network: (**a**) Li@G3, (**b**) TFSI, (**c**) MDA, and (**d**) DGEBA. Color code: O atoms are in red, C: gray, H: white, Li: purple, S: yellow, N: blue, and F: aqua.

The atomic structures of the components of the polymer network were created using the software package GaussView. Initially, all structures were optimized using the universal force field (UFF) as implemented in GaussView [14,15], and then the density functional theory (DFT) calculation level of B3YLP/6-31g(d) was used, whereby tight convergence criteria were applied for geometry optimization, and frequency calculations were conducted as implemented in Gaussian 16 [16,17,18]. Figure 5 illustrates the initial atomic structures of the 3D crosslinked network.

- Reacting Molecules

Identifying the right activated ends in the reaction molecules of the 3D crosslinked network may become challenging when running molecular dynamic simulations to predict innovative material properties [10]. Here, other approaches are required to identify the right configuration of the SIL and to investigate its effect on the rapid curing of the thermoset polymers.

**Figure 6:**
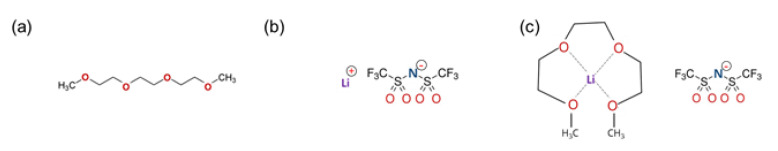
A schematic diagram illustrating the mechanism of the formation of the solvate ionic liquid: (**a**) triglyme (G3) and (**b**) a group 1A cation with a counterion apart from TFSI, forming (**c**) [Li@G3]^+^ [TFSI].

Before understanding how the SIL increases the curing rates, it is imperative to understand how the TFSI-based additive is formed. Generally, in an equimolar mix, a group of 1A cation (i.e., Li) and a glyme-like chelating agent (i.e., Triglyme) tend to coordinate to form one-to-one complexes of [Li@G3]^+^ in the ionic liquid of [TFSI]^−^ [19,20], as illustrated in Figure 6.

**Figure 7:**
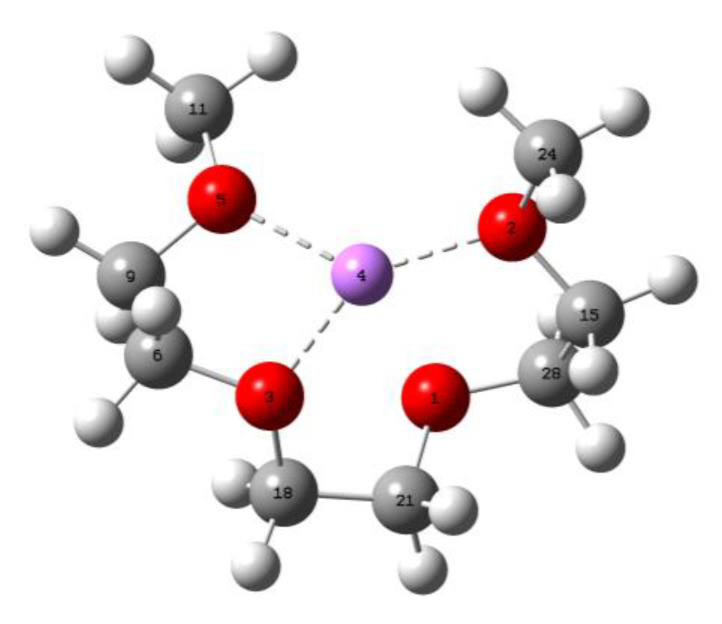
[Li@G3]^+^ showing three oxygen bonds with lithium.

A quantum mechanics approach was followed to confirm the atomic structure of [Li@G3]^+^. We applied the Møller–Plesset truncated expansion at the second order (MP2) level of the calculation method with a def2tzvp basis set as implemented in Gaussian 16.0, using the initially optimized geometry as demonstrated in Figure 5a and Figure 6c [21,22]. The optimized geometry showed that G3 coordination is high, and three oxygen atoms to Li^+^ are constructed in the equimolar liquid, which agrees with the study conducted by Shimuzu, as demonstrated in Figure 7 [23]. Shimuzu applied molecular dynamics using the OPLS force field to predict that, on average, only three oxygen atoms of the 152 triglyme molecules out of 495 bind to lithium-ion, whereas we applied quantum mechanics as described above. Table 1 summarizes the Li-O bond length, bond angles, and dihedral angles. The optimized geometry was verified using normal mode analysis (i.e., no imaginary frequencies).

**Figure 8:**
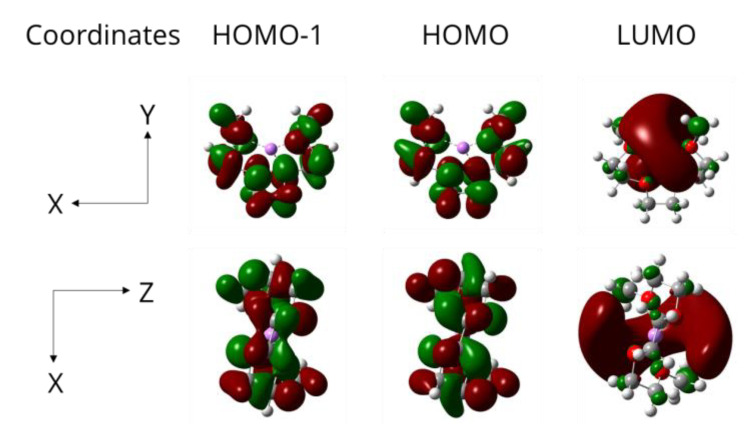
HOMO-1, HOMO, and LUMO of [Li@G3]^+^ generated using GaussView at Isovalue of 0.025.

The spatial distributions of the HOMO, HOMO-1, and LUMO of the complex are presented in Figure 8. The HOMO and HOMO-1 are localized around the lithium atom, and the LUMO is localized around the ligand. Additionally, the results confirm that spatial distributions of HUMO and HUMO-1 are similar. This reveals the high tendency of the lithium atom in the ligand to create a bond with an oxygen atom if placed within the (Li-O) bond length. The lithium-ion is a hard Lewis acid, and the triglyme is a hard Lewis base because of the presence of lone pair orbitals on the oxygen atoms either at the glyme or the DGEBA, as confirmed by Yoshida and Dagdag, respectively [24,25].

**Figure 9:**
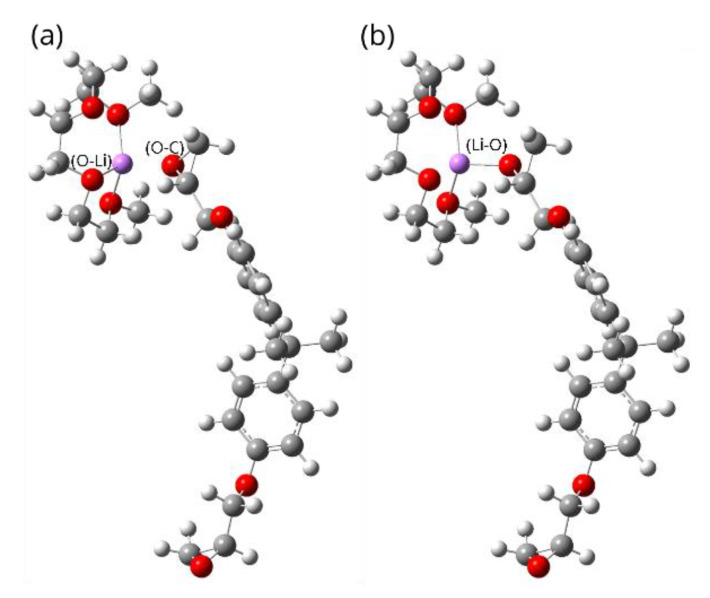
[Li@G3]^+^ and DGEBA. (**a**) The reactant complex (RC) where no ring is opened and the Li-O bond has not been created. (**b**) Product complex (PC) where the ring is opened and the Li-O bond is created.

Subsequently, we utilized the final optimized structure of [Li@G3]^+^ to investigate how the formed SIL, particularly [Li@G3]^+^ [TFSI]^−^, will react in the presence of epoxy resin (i.e., DGEBA/MDA). The structures of the reactant complex (RC) and the product complex (PC) were created as follows: The previously optimized geometries of the DGEBA and [Li@G3]^+^ were first frozen. Then, several rigid scans of single-point energy between the lithium atom and the oxygen atom at the DGEBA ring were implemented via Gaussian 16.0 using different configurations, as explained elsewhere [17,26]. Once the lowest configuration produces the lowest energy level, the structure is reoptimized without any constraints. All calculations utilized the DFT level of M062X/6-311++G (2d, 2p) [27,28]. Figure 9 depicts RC and PC.

**Figure 10:**
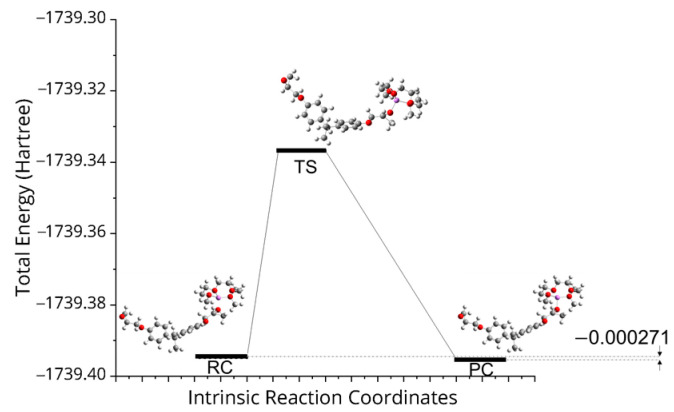
TS, RC, and PC complexes.

**Figure 11:**
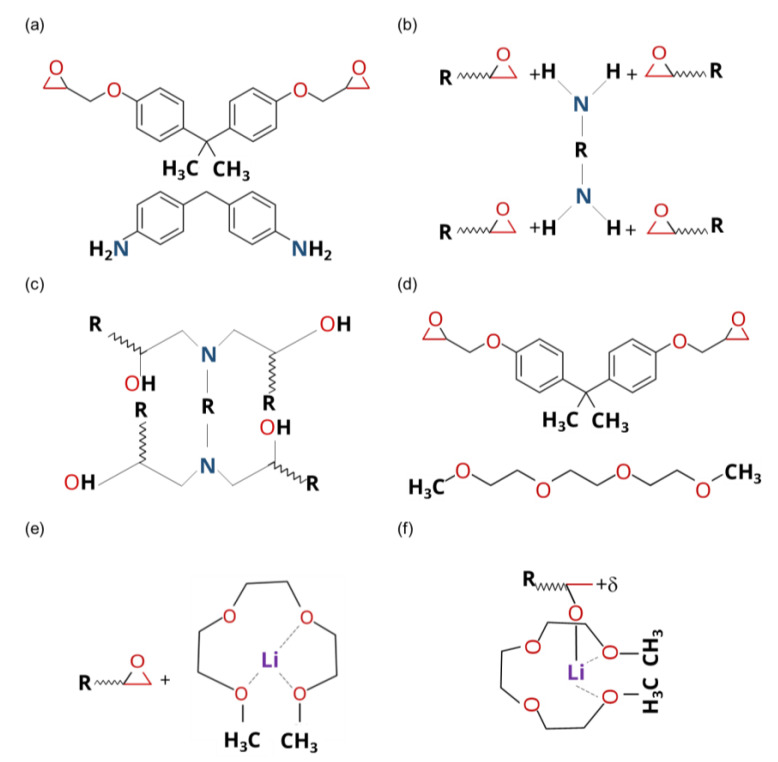
Reactive molecules and interaction mechanisms: (**a**) structures of the epoxy–amine combination, (**b**,**c**) interaction of the epoxy–amine, (**d**) structures of SIL and the epoxy, and (**e**,**f**) interaction of the metal atom in the SIL chelate.

**Figure 12:**
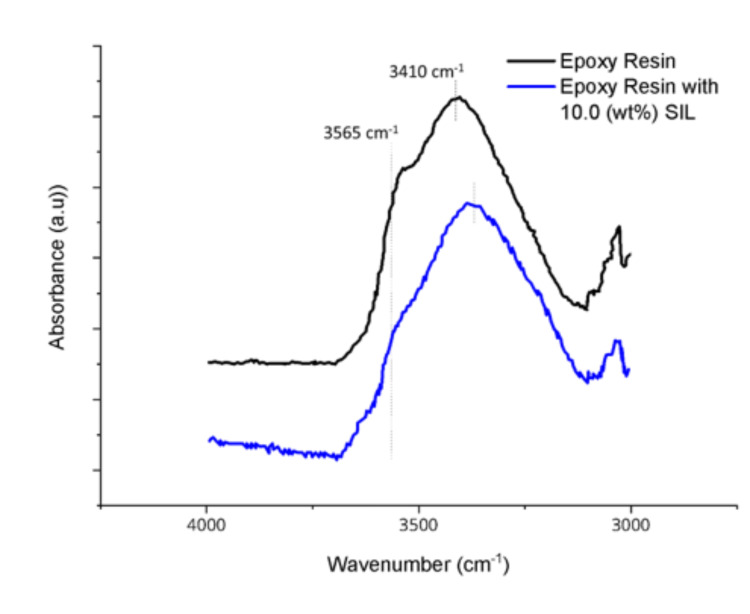
The hydroxyl stretching region in infrared spectra of epoxy resin with and without 10.0 wt. SIL.

We utilized the synchronous transit guided quasi-Newton methods to search transit states (TSs) using RC and PC [29]. Then, the intrinsic reaction coordinates (IRCs) method from the transition states was applied as implemented in Gaussian 16.0 to ensure that each resulting saddle point was associated with its corresponding RC and PC [30]. All obtained geometries were verified by applying normal mode analysis to be minima (i.e., all real frequencies) or maxima (i.e., one imaginary frequency). Figure 10 presents the reaction of RC and PC complexes. The transition state confirms the high coordination of Li^+^ and O atoms, the formation of the new complexes, and the bonding structure associated with them, as presented in Figure 11 and evident in the FTIR analysis (Figure 12). In neat cured epoxy resin, two distinctive bands at 3410 cm^−1^ and 3565 cm^−1^ can be attributed to the stretching vibrations of self-associated hydroxyl groups and non-associated free hydroxyl groups, respectively. With the presence of the 10.0 wt.% SIL, the peak attributed to the hydroxyl stretching band at 3410 cm^−1^ is shifted to a lower frequency, implying the formation of the new complexation. Still, the peak corresponding to the free hydroxyl groups disappears.

- Partial Charges

Reacted forms of atoms’ initial charges were calculated in vacuo using the RESP ESP charge drive (RED) methodology [31]. This methodology accurately estimates charges using quantum-mechanics-theory-level calculations for geometry optimization and charge distribution. In return, the theory-level calculations allow for the checking of the reliability using vibrational analysis. The Li atom in the triglyme ligand was modeled, and its charge and force field parameters were calculated using the metal center parameter builder (MCPB) tool as implemented in the assisted model building with energy refinement (AMBER 2020) tool [32]. Other atoms’ charges were calculated using the general force field (GAFF) as implemented in AMBER 2020 [33,34]. All missing force field parameters were defined by AMBER 2020, too [32]. These methodologies were carefully chosen for their high accuracy, reliability, and reproducibility [35]. The special bond types created between O atoms in the triglyme ligand and Li are illustrated in Figure 11. Initial charges of the molecular configurations for the molecular dynamics set are listed in Tables S1 and S2.

- 3D Structure

Simulation boxes were created using the Packmol software package, where initial molecular configurations in stoichiometric ratios were randomly packed in cubic cells [36]. Since the liquid mixtures of DGEBA, MDA, and SIL crosslink rapidly to form a solid 3D network, there is no experimental benchmark for the change in the density and structure of the mixture that we can validate and use to validate our force field parameters further. On the contrary, available relative density measures are mostly at room temperature (i.e., 298.0 k), where the densities of DGEBA and MDA are around 1.17 g cm^−3^ and 1.07 g cm^−3^, respectively. The density of the SIL is estimated to be around 1.472 g cm^−3^ [23]. Therefore, the stoichiometric ratio between DGEBA and MDA was adopted and kept at 100 to 28, respectively. Furthermore, the simulation adopted and considered a 10.0 wt.% of an equimolar mixture of Li(TFSI) and G3. Thus, the mixture’s predicted density could not exceed 1.17 g cm^−3^ around room temperature, indicating the initial volume and initial simulation cell, which can reach the required liquid density well above curing. Several approaches were applied to establish the density and structure of the pure liquid of the reacted mixtures in the literature [37]. Since this system includes the SIL structure, establishing the density while fine-tuning the force field parameters may become challenging. Therefore, other simulation parameters need to be considered, including time-step simulation. In return, the rapid nature of such crosslinking requires several iterations of changing molecular dynamics stimulation parameters to reach the equilibrated structure, which we will discuss in the following section.

- 3D Equilibrated and Crosslinked Structures

**Figure 13:**
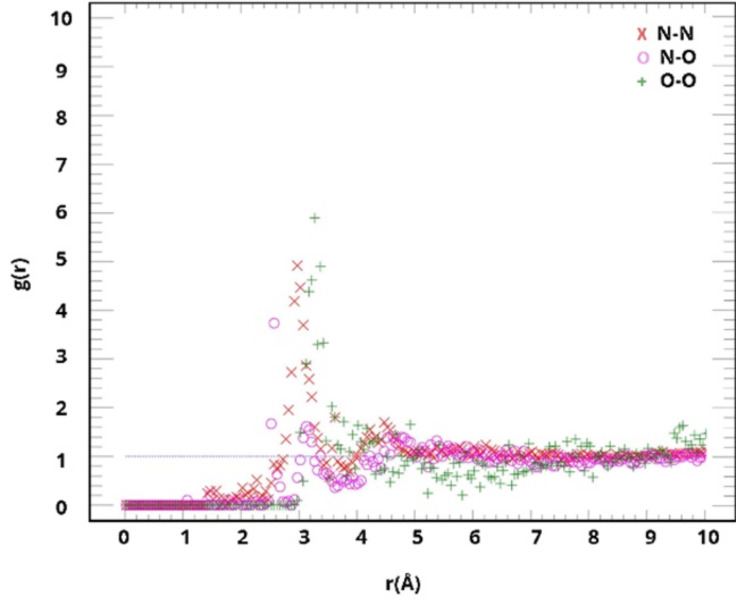
Evaluation of the average RDFs of the six simulated annealing cycles calculated for the liquid mixture.

The reaction mixture is large; hence it is crucial to ensure that the system is well-mixed. Therefore, the reaction mixture must be at equilibrium before crosslinking since an imbalanced reaction mixture can lead to inaccurate predictions. This was achieved, first, by creating a unit cell of the unreacted mixture and then bringing it to the minimum energy using the fast internal relaxation engine (Fire), followed by the Polak–Ribiere version of the conjugate gradient (CG) as implemented in the Large-scale Atomic/Molecular Massively Parallel Simulator (LAMMPS) software package 2020 [38,39,40]. The Ewald algorithm was applied to calculate the columbic interactions with a real-space cutoff of 12 Å [41]. The velocity–Verlet integrator was applied to integrate the equation of motions using a time-step of 1 fs [42], and the temperature and pressure were regulated using the Nosé–Hoover thermostat and barostat [43,44]. Three cells of the reaction mixture of 60.0 × 64.0 × 64.0 Å^3^ were created using Packmol with 16,160 atoms each. Periodic boundary conditions were implemented in all three dimensions. Each system was initially equilibrated with six annealing cycles from 300 to 500 °K for 20 ps and optimized, after each cycle, to reach the minimum potential energy level. This was crucial for reducing the total net forces on atoms and confirming that the force field parameters accurately regenerated experimental values for continuum-level properties or quantum mechanical value geometries [37]. In return, this ensured statistical significance and avoided bias. Subsequently, the equilibration of the three samples was evaluated using the radius distribution functions (RDFs) as a function of the simulated annealing cycle. The resulting average of the RDFs calculated after the six simulated annealing cycles is illustrated in Figure 13. The six cycles’ RDFs for each sample are presented in Figure S1. The radii distribution between Nitrogen–Nitr–gen, Oxygen–Oxygen, and Nitrogen–Oxygen illustrates the distribution of MDA-MDA, DGEBA-DGEBA, and MDA-DGEBA, respectively. The first peak in the distributions of molecules was mostly located at around 3.0 Å, whereas the MDA-DGEBA distribution was at around 4.5 Å.

- 3D Crosslinking Procedure

**Figure 14:**
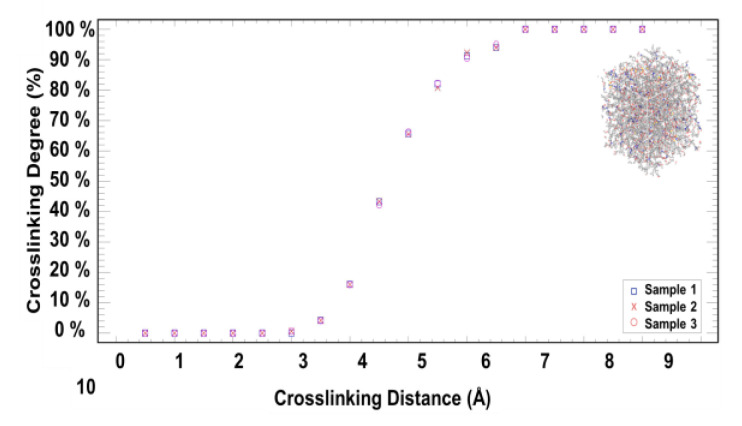
The percentage of the crosslinking degree of the three reaction mixtures as a function of crosslinking distance.

Following equilibration, the crosslinking procedure was applied, covering a cutoff distance between the reactive sites starting from 3.0 to 7.0 Å with an increment of 0.5 Å, as indicated in Figure 14. Crosslinking for each run occurred for all molecules whose reactive sites were within the cutoff distance, say 3.0 Å. By following each attempt of crosslinking with a set of relaxation and optimization of the polymeric networks, we considered the self-ring-opening mechanisms associated with SIL using NVT and NPT molecular dynamic simulations to allow diffusion, bringing reactive atoms closer to each other for the next attempt. For each relaxation cycle, the temperature was increased in steps of 50 °K for 20 ps each from 300 °K to 500 °K, and a recheck was performed should more crosslinks be created with the cutoff distance (e.g., 3 Å). The procedure was repeated until nothing changed, and the targeted crosslinking level was achieved.

In the beginning, the crosslinking is relatively high, as measured by the change in the crosslinking degree over the cutoff distance—more than 65.0% of the reactive atom pairs were crosslinked at a 5.0 Å cutoff distance or less. Then, the crosslinking degree increases. The high crosslinking degree at a relatively short cutoff distance is mainly due to the reactive atom pairs resulting from the SIL. The variability among the three crosslinked samples is relatively low, suggesting that the three reaction mixtures are well-mixed and equilibrated.

At a 5.5 Å cutoff distance, corresponding to an 81.0% crosslinking degree, the average crosslinking bond length of the three samples after equilibration was measured and found to be 1.474 ± 0.003 Å. Likewise, the average crosslinking bond angle was measured and found to be 110.634o ± 1.138o (Figure S2). When calculated using the AMBER force field, the ideal values were 1.46 Å and 112.45o, respectively. These crosslinking bond values are statistically comparable to their corresponding AMBER force field values at an error value of less than 1.0%. The internal pressure of the three crosslinked samples was calculated and verified to ensure it was stress-free (Figure S3). The results show acceptable fluctuation levels around the targeted pressure of 1.0 × 10^−4^ GPa (i.e., 1 atm), which is expected under the isothermal-isobaric (NPT) ensemble in molecular dynamics.

- Prediction of Continuum-Level Properties

The three crosslinked samples with a crosslinking degree of 91.0%, associated with the 6.0 Å cutoff distance, were considered to predict the thermo-mechanical properties using the output of the isobaric cooling simulation. These included the glass transition temperature, the coefficient of the volumetric thermal expansion (CVTE) (*α_v_*), and the coefficient of linear thermal expansion (CLTE) (*α_l_*). *α_v_* and *α_l_* were predicted using Equations (1) and (2):

(1) $$\alpha_{v}=\frac{1}{V_{o}}{\left(\frac{\partial V}{\partial T}\right)}_{P}$$

(2) $$\alpha_{l}=\frac{\alpha_{v}}{3}$$

where

*V_o_* is the initial volume at the initial temperature;

$\frac{\partial V}{\partial T}$ is the change in volume concerning temperature. 

Each sample was heated to 500 °K and then cooled to 300 °K; then, the density and energy were averaged over the production run. At 300 °K, the density was 1.08 ± 0.01 g/cm^−3^ and was in good agreement with experimental values of around 1.1 ± 0.01 g/cm^−3^. Three crosslinked samples with a crosslinking degree of 91.0% were utilized to predict mechanical properties, particularly material behavior under continuous stress and Young’s modulus. Uniaxial tensile simulations were performed to predict the mechanical behavior of the amorphous structure. The Parrinello–Rahman fluctuation strain method was applied using the NσT ensemble [45] at a temperature of 300 °K while applying incremental stress of 0.05 GPa until reaching 2.5 GPa. Each simulation run was set for 100.0 ps, and the obtained result for each run was averaged over the three samples

## 3. Results and Discussion

**Figure 15:**
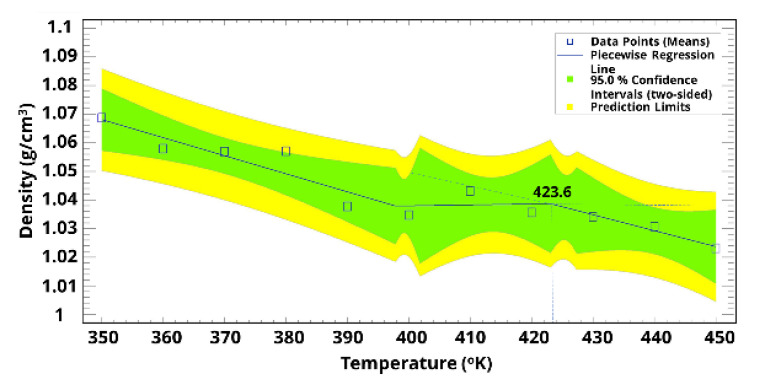
Prediction of the Tg using system density as a function of temperature using piecewise regression.

**Figure 16:**
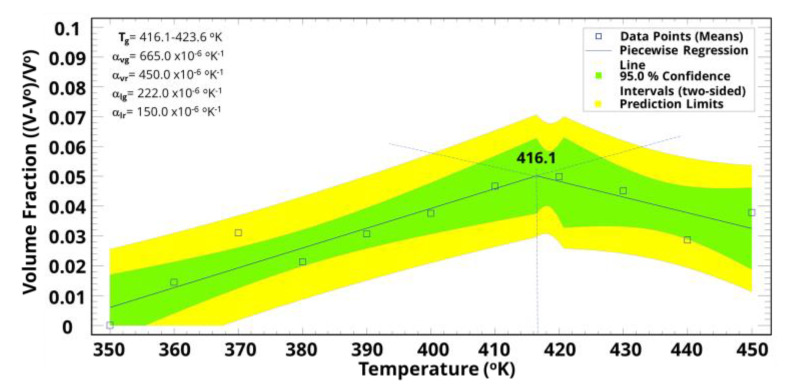
Prediction of some thermo-mechanical properties using piecewise regression based on the volume fraction as a function of temperature.

Following the molecular dynamic simulation, Tg was determined using piecewise regression to statistically identify the phase transition between glass and rubber, as indicated in Figure 15 and Figure 16. The predicted Tg was between 416.3 °K and 423.1 °K, and these values agree with our previous work [1] and current experimental work that showed a Tg value of around 417.0 ± 1.0 °K. Furthermore, the volumetric thermal expansion coefficient at the glass phase was around 1.5-fold that of the rubber phase, suggesting a rapid cure due to the sudden change around the Tg.

**Figure 17:**
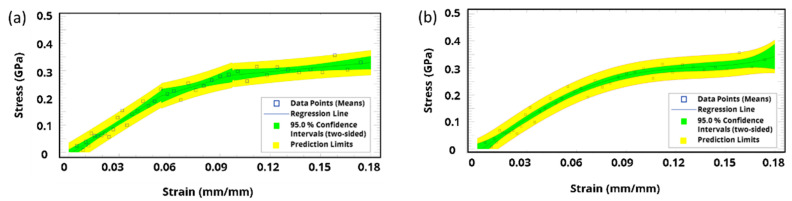
Stress–strain curve constructed from the molecular dynamics: (**a**) piecewise regression to investigate mechanical behavior regions, and (**b**) polynomial-based fitted model of the stress–strain curve.

Likewise, the molecular dynamics simulation results based on the Parrinello–Rahman fluctuation strain method were utilized to construct the stress–strain curve. Piecewise regression was initially used to investigate mechanical behavior regions, as illustrated in Figure 17a: three regions can be distinguished, starting from the elastic region, and then the plateau, and finally the fracture. Unlike thermosets that are generally brittle and cannot be stretched beyond the elastic region, our obtained results of the molecular dynamic simulations were confirmed via our experimental data [46]. Stress–strain data were fitted with a polynomial curve using a least squares method to determine yield stress and Young’s modulus. Raw data from the molecular dynamics simulation are presented in Figure 17b along with the fitted curve. Subsequently, the polynomial-based model was fitted again using stepwise regression (i.e., elastoplastic), applying two bilinear lines with the least squares model. The general stress–strain fitting objective function (Equation (3)) aims to minimize the stress residue. The fitted value becomes the closest to the real value under a certain confidence level (e.g., 95.0%).

(3) $$r\left(\sigma ,\varepsilon ,k\right)=\sigma_{y}-f\left(\sigma ,\varepsilon ,k\right)\;$$

where:

σ is the value of the fitted stress resulting from the molecular dynamics simulation;

ε is the strain;

$\sigma_{y}$ is the yield stress;

k is the hardening parameter;

$f\left(\sigma ,\varepsilon ,k\right)\;$is the fitted curve.

**Figure 18:**
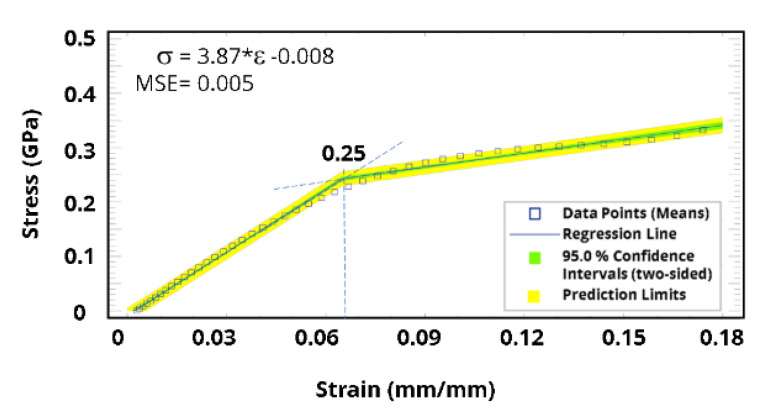
A criterion for the yield point obtained from the fitted stress–strain response, depicting the analysis at a crosslinking degree of 91.0%.

**Figure 19:**
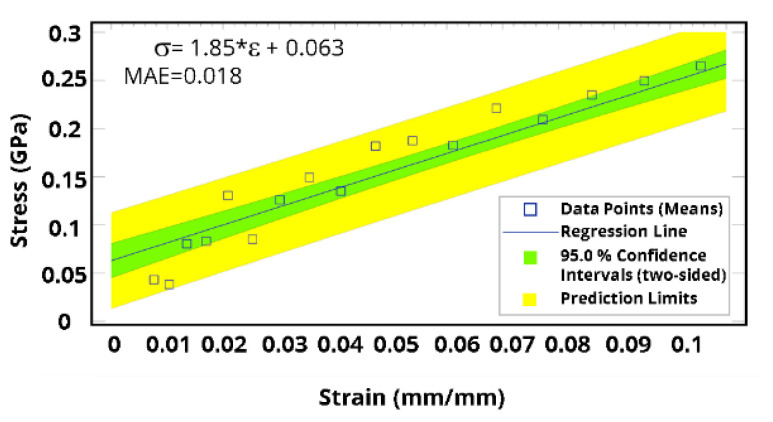
A criterion for the yield point, depicting the analysis at a crosslinking degree of 43.0%.

Building on the fitted model, Young’s modulus, yield stress, and hardening parameters were calculated (Figure 18). At 91.0% crosslinking degree, the yield stress and Young’s modulus were estimated to be around 250 MPa and 3.865 GPa, respectively. Since yield stress and Young’s modulus are directly proportional to the crosslinking degree of 39, additional molecular dynamics simulations were conducted to investigate experimental work using a lower crosslinking degree. Figure 19 shows the analysis results when applying a crosslinking degree of 43.0%, where Young’s modulus is around 1.85 GPa, which is in good agreement with our experimental work.

The curing regime, covering mixing ratio, and curing time, to name a few, can affect the microstructure, which can directly affect macro properties, especially the thermomechanical ones [47]. Since the weight percent of the SIL strongly influences the crosslinking reaction kinetics, the effect of the mixing ratio can be insignificant due to the rapid curing nature of this unique system [1]. Nevertheless, this nature may lead to inconsistency in predicting the continuum-level properties, which we accounted for in our protocol, along with others, increasing the system size to more than 16,000 atoms, maintaining a relatively high cycle time in hundreds of picoseconds while reducing the step rate to 1 fs or 1 fs and 0.05 GPa, and setting a crosslinking degree of 90.0% or above. These setups are aligned with well-performing models, confirming that the larger the model size is (e.g., 15,000 atoms), the more accurate the outcome will be [48,49]. Additionally, increasing the cycle time while reducing the step rate can lead to consistent yet accurate outcomes if higher than the under-study reaction time [50]. Likewise, setting a uniform crosslinking degree of 90% or above can reduce inconsistency and increase accuracy significantly [48,49]. Hence, this protocol can effectively strike a balance between reliable and reproducible results, confirmed by experimental work, and a relatively low computational cost.

## 4. Conclusions

A novel protocol to predict thermo-mechanical properties of rapid curing thermosets was developed based on quantum mechanics and molecular dynamics. The quantum mechanics approaches were applied to identify the epoxy resin’s reactive sides in the presence of the SIL. Furthermore, quantum mechanics was applied to determine initial partial atomic charges and force field parameters apart from RED methodology. The equilibration of the liquid precursor mixture and the three-dimensional crosslinking approach using statistically representative samples have proven to be reliable, especially when the obtained results were used to predict thermo-mechanical properties regarding glass transition temperature coefficients of the volumetric thermal expansion and linear thermal expansion. The Parrinello–Rahman fluctuation strain method was applied to predict mechanical behaviors using the NσT ensemble. Statistical techniques based on piecewise regression and linear and polynomial regression models were implemented to establish several yield stress criteria and predict Young’s modulus at different crosslinking degrees. This protocol makes the molecular dynamics simulation more reliable for polymeric material design, especially when considering any additives to enhance the thermo-mechanical properties.

## Figures and Tables

**Table 1:** Summary of bond lengths and bond/dihedral angles of [Li@G3]^+^ resulting from the quantum mechanics modeling with atom numbers (Figure 7).

S/N	Bond Length (Å)	O-Li-O Angle (^o^)	O-C-C-O Angle (^o^)
1	5-4 (1.95226)	5-4-3 (85.81815)	5-9-6-3 (−47.59083)
2	3-4 (1.99950)	3-4-2 (129.63429)	3-18-21-1 (−58.34169)
3	2-4 (1.95226)	5-4-2 (135.18919)	1-28-15-2 (−47.59071)

## Data Availability

The datasets generated during and/or analysed during the current study are available from the corresponding author on reasonable request.

## Supplementary Materials

The following supporting information can be downloaded at https://www.mdpi.com/article/10.3390/polym15051325/s1: Table S1:Atom charges of molecules used in the computational modeling based on RED methodology. Table S2: Atom charges of Li@3G with DGEBA used in the computational modeling based on MCPB methodology. Figure S1: Evaluation of the RDFs calculated for the liquid mixture as a function of the six simulated annealing cycles. Figure S2: Bond lengths and angles of the crosslink bonds for the equilibrated/crosslinked samples at 81.0% crosslinking degree. Figure S3: Calculated internal pressure of the sample after the crosslinking process.
