# Supplementary material for: Crosslinking Rapidly Cured Epoxy Resin Thermosets: Experimental and Computational Modeling and Simulation Study
Source: Polymers (Basel). 2023 Mar 6;15(5):1325. doi: 10.3390/polym15051325 (PMC10007365; doi:10.3390/polym15051325)
Supplement: Supplementary file 1 [file polymers-15-01325-s001.zip › polymers-2227626-supplementary.pdf]

Supporting Information

Crosslinking rapidly cured epoxy resin thermosets: experimental and computational modeling and simulation study

Ahmed Al-Qatatsheh,[a] Jaworski C. Capricho,[a] Paolo Raiteri,[b] Saulius Juodkazis,[a,c] Nisa Salim[a], and Nishar Hameed*[a]

[a] School of Engineering, Swinburne University of Technology, Melbourne VIC 3122, Australia.

[b] School of Molecular and Life Sciences, Faculty of Science and Engineering, Curtin University, Perth WA 6845, Australia.

[c] Optical Sciences Centre and ARC Training Centre in Surface Engineering for Advanced Materials (SEAM), Swinburne University of Technology, Melbourne VIC 3122, Australia.

* E-mail: nisharhameed@swin.edu.au

Table of Contents

Table S1: Atom charges of molecules used in the computational modeling based on RED. Methodology. **3**

Table S2: Atom charges of Li@3G with DGEBA used in the computational modeling based on MCPB Methodology**.** **3**

Figure S1: Evaluation of the RDFs calculated for the liquid mixture as a function of the six simulated annealing cycles. **5**

Figure S2: Bond lengths and angles of the crosslink bonds for the equilibrated/crosslinked samples at 81.0 % crosslinking degree. **5**

Figure S3: Calculated internal pressure of the sample after the crosslinking process. **5**

Table S1. Atom charges of molecules used in the computational modeling based on RED Methodology.

TFSI			MDA			DGEBA					
S/N	Atom	Charge	S/N	Atom	Charge	S/N	Atom	Charge	S/N	Atom	Charge
1	**C1**	**0.4438**	1	**C1**	**-0.1871**	1	**O1**	**-0.3533**	30	**H30**	**0.1002**
2	**S2**	**0.7571**	2	**H2**	**0.1892**	2	**O2**	**-0.3502**	31	**H31**	**0.1002**
3	**F3**	**-0.1292**	3	**C3**	**-0.2885**	3	**O3**	**-0.3949**	32	**C32**	**-0.1314**
4	**F4**	**-0.1370**	4	**H4**	**0.1712**	4	**O4**	**-0.3757**	33	**H33**	**0.1547**
5	**F5**	**-0.1489**	5	**C5**	**0.3879**	5	**C5**	**0.1616**	34	**C34**	**-0.1614**
6	**N6**	**-0.7125**	6	**C6**	**-0.2988**	6	**C6**	**-0.0068**	35	**H35**	**0.1585**
7	**O7**	**-0.4851**	7	**H7**	**0.1818**	7	**H7**	**0.1440**	36	**C36**	**-0.1602**
8	**O8**	**-0.4954**	8	**C8**	**-0.1933**	8	**C8**	**-0.0237**	37	**H37**	**0.1658**
9	**S9**	**0.9449**	9	**H9**	**0.1544**	9	**H9**	**0.1496**	38	**C38**	**-0.1638**
10	**C10**	**0.2922**	10	**C10**	**0.0283**	10	**C10**	**-0.0394**	39	**H39**	**0.1626**
11	**O11**	**-0.5247**	11	**C11**	**-0.2598**	11	**C11**	**-0.0215**	40	**C40**	**0.3364**
12	**O12**	**-0.5101**	12	**H12**	**0.1729**	12	**C12**	**-0.0827**	41	**C41**	**0.2898**
13	**F13**	**-0.0932**	13	**C13**	**-0.2202**	13	**H13**	**0.1285**	42	**C42**	**-0.2535**
14	**F14**	**-0.1339**	14	**H14**	**0.1613**	14	**H14**	**0.1285**	43	**H43**	**0.1527**
15	**F15**	**-0.0680**	15	**C15**	**0.0378**	15	**C15**	**-0.072**	44	**C44**	**-0.2123**
			16	**C16**	**-0.1988**	16	**H16**	**0.1261**	45	**H45**	**0.1445**
			17	**H17**	**0.1908**	17	**H17**	**0.1261**	46	**C46**	**-0.2478**
			18	**C18**	**-0.2633**	18	**C18**	**-0.1556**	47	**H47**	**0.1107**
			19	**H19**	**0.1677**	19	**H19**	**0.0422**	48	**C48**	**-0.2266**
			20	**C20**	**0.3462**	20	**H20**	**0.0422**	49	**H49**	**0.1086**
			21	**C21**	**-0.0255**	21	**H21**	**0.0422**			
			22	**H22**	**0.0407**	22	**C22**	**-0.156**			
			23	**H23**	**0.0407**	23	**H23**	**0.0419**			
			24	**N24**	**-0.8843**	24	**H24**	**0.0419**			
			25	**H25**	**0.3604**	25	**H25**	**0.0419**			
			26	**H26**	**0.3604**	26	**C26**	**0.1102**			
			27	**N27**	**-0.8859**	27	**H27**	**0.0932**			
			28	**H28**	**0.3569**	28	**H28**	**0.0932**			
			29	**H29**	**0.3569**	29	**C29**	**0.0908**			

Table S2. Atom charges of Li@3G with DGEBA used in the computational modeling based on MCPB Methodology.

**Li@G3 with DGEBA**											
S/N	Atom	Charge	S/N	Atom	Charge	S/N	Atom	Charge	S/N	Atom	Charge
1	**C1**	**-0.0982**	21	**H21**	**0.0567**	41	**C41**	**0.3744**	61	**O61**	**-0.4076**
2	**H2**	**0.0835**	22	**H22**	**0.0567**	42	**C42**	**-0.362**	62	**C62**	**0.2658**
3	**H3**	**0.0835**	23	**C23**	**0.0202**	43	**H43**	**0.0855**	63	**H63**	**0.0017**
4	**H4**	**0.0835**	24	**H24**	**0.0633**	44	**H44**	**0.0855**	64	**H64**	**0.0017**
5	**O5**	**-0.1628**	25	**H25**	**0.0633**	45	**H45**	**0.0855**	65	**C65**	**0.0855**
6	**C6**	**-0.0621**	26	**O26**	**-0.2566**	46	**C46**	**-0.3668**	66	**H66**	**0.0231**
7	**H7**	**0.0756**	27	**C27**	**-0.1253**	47	**H47**	**0.0868**	67	**H67**	**0.0231**
8	**H8**	**0.0756**	28	**H28**	**0.0883**	48	**H48**	**0.0868**	68	**C68**	**0.4944**
9	**C9**	**0.0276**	29	**H29**	**0.0883**	49	**H49**	**0.0868**	69	**H69**	**-0.036**
10	**H10**	**0.0672**	30	**H30**	**0.0883**	50	**C50**	**-0.2351**	70	**C70**	**0.5474**
11	**H11**	**0.0672**	31	**C31**	**-0.0918**	51	**H51**	**0.1505**	71	**H71**	**-0.0795**
12	**O12**	**-0.1681**	32	**H32**	**0.118**	52	**C52**	**-0.1666**	72	**O72**	**-0.4436**
13	**C13**	**-0.0966**	33	**C33**	**-0.3029**	53	**H53**	**0.1185**	73	**O73**	**-0.846**
14	**H14**	**0.0814**	34	**H34**	**0.1494**	54	**C54**	**0.006**	74	**C74**	**-0.4199**
15	**H15**	**0.0814**	35	**C35**	**0.3819**	55	**C55**	**-0.181**	75	**H75**	**0.1096**
16	**C16**	**0.1147**	36	**C36**	**-0.2562**	56	**H56**	**0.1227**	76	**H76**	**0.1096**
17	**H17**	**0.0616**	37	**H37**	**0.1187**	57	**C57**	**-0.2255**	77	**C77**	**-0.1992**
18	**H18**	**0.0616**	38	**C38**	**-0.1242**	58	**H58**	**0.1396**	78	**H78**	**0.0703**
19	**O19**	**-0.3474**	39	**H39**	**0.1282**	59	**C59**	**0.3548**	79	**H79**	**0.0703**
20	**C20**	**0.0542**	40	**C40**	**-0.0636**	60	**O60**	**-0.3797**	80	**Li1**	**0.4783**

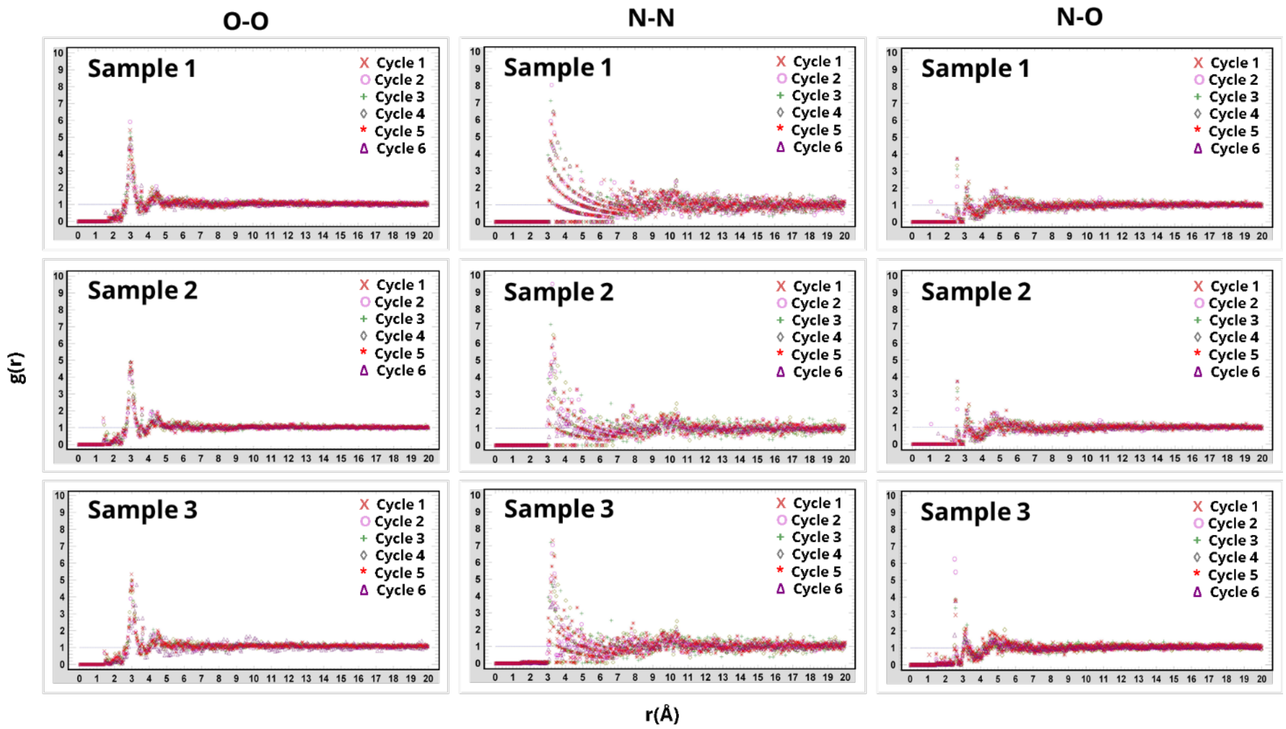

Figure S1. Evaluation of the RDFs calculated for the liquid mixture as a function of the six simulated annealing cycles.

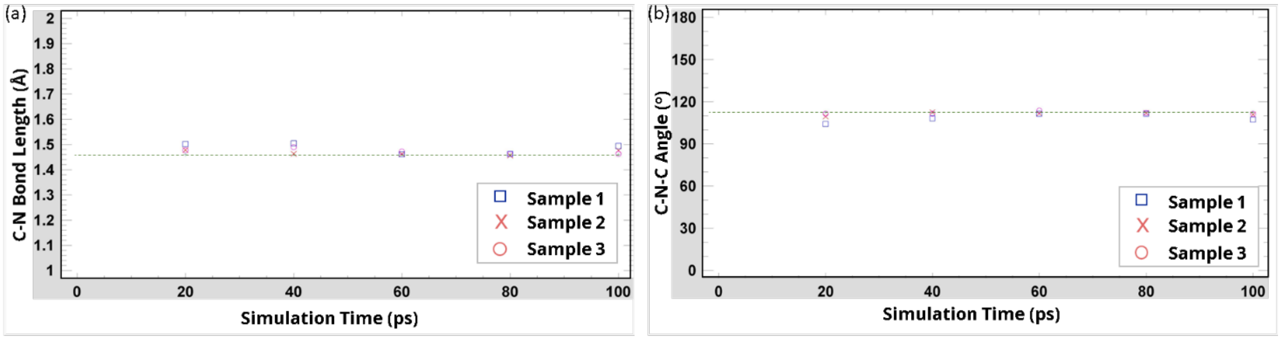

Figure S2. (**a**) Bond lengths of the crosslink bonds, and (**b**) bond angles between the C-N-C atoms of the crosslink bonds for equilibrated crosslinked samples at 81.0 % crosslinking degree. The dashed lines represent the AMBER Force Field ideal values.

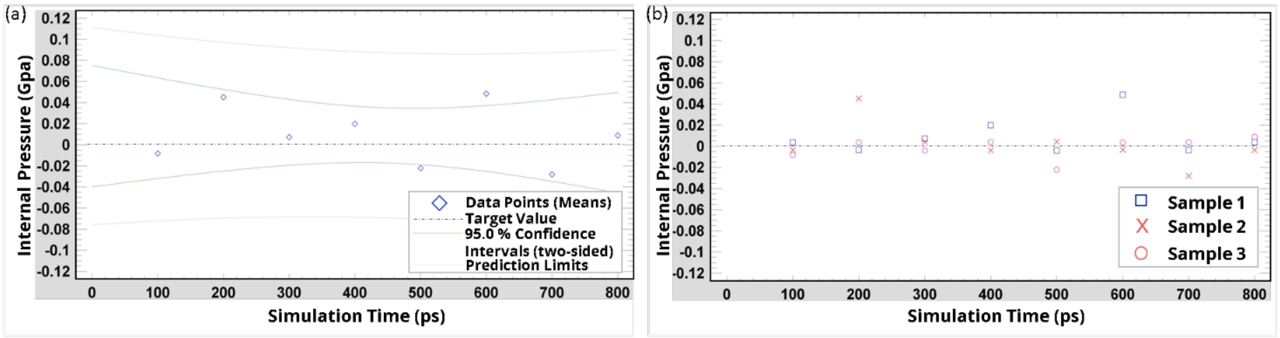

Figure S3. (**a**) Calculated average internal pressure of the sample after the crosslinking process, and (**b**) the internal pressure of the three crosslinked samples. The dashed line represents the target pressure, 0.0001 GPa.
